# The challenge of improving long-lasting insecticidal nets coverage on Bioko Island: using data to adapt distribution strategies

**DOI:** 10.21203/rs.3.rs-4188387/v1

**Published:** 2024-04-05

**Authors:** Guillermo A. García, David S. Galick, Jordan M. Smith, Marcos Mbulito Iyanga, Matilde Riloha Rivas, Jeremías Nzamío Mba Eyono, Wonder P. Phiri, Olivier Tresor Donfack, David L. Smith, Carlos A. Guerra

**Affiliations:** 1 MCD Global Health, 8403 Colesville Road, Suite 425, 20910 Silver Spring, USA.; 2 MCD Global Health, Av. Parques de Africa, Malabo, Equatorial Guinea.; 3 National Malaria Control Program, Ministry of Health and Social Welfare of Equatorial Guinea, Malabo, Equatorial Guinea.; 4 Institute for Health Metrics and Evaluation, University of Washington, 2301 Fifth Avenue, 98121 Seattle, USA.

**Keywords:** long-lasting insecticidal nets, mass-distribution campaign, malaria, malaria indicator survey, LLIN indicators, coverage

## Abstract

**Background:**

Since 2015, malaria vector control on Bioko Island has relied heavily upon long-lasting insecticidal nets (LLIN) to complement other interventions. Despite significant resources utilised, however, achieving and maintaining high coverage has been elusive. Here, core LLIN indicators were used to assess and redefine distribution strategies.

**Methods:**

LLIN indicators were estimated for Bioko Island between 2015 and 2022 using a 1×1 km grid of areas. The way these indicators interacted was used to critically assess coverage targets. Particular attention was paid to spatial heterogeneity and to differences between urban Malabo, the capital, and the rural periphery.

**Results:**

LLIN coverage according to all indicators varied substantially across areas, decreased significantly soon after mass distribution campaigns (MDC) and, with few exceptions, remained consistently below the recommended target. Use was strongly correlated with population access, particularly in Malabo. After a change in strategy in Malabo from MDC to fixed distribution points, use-to-access showed significant improvement, indicating those who obtained their nets from these sources were more likely to keep them and use them. Moreover, their use rates were significantly higher than those of whom sourced their nets elsewhere.

**Conclusions:**

Striking a better balance between LLIN distribution efficiency and coverage represents a major challenge as LLIN retention and use rates remain low despite high access resulting from MDC. The cost benefit of fixed distribution points in Malabo was deemed significant, providing a viable alternative for guaranteeing access to LLINs to those who use them.

## Background

**Figure 1:**
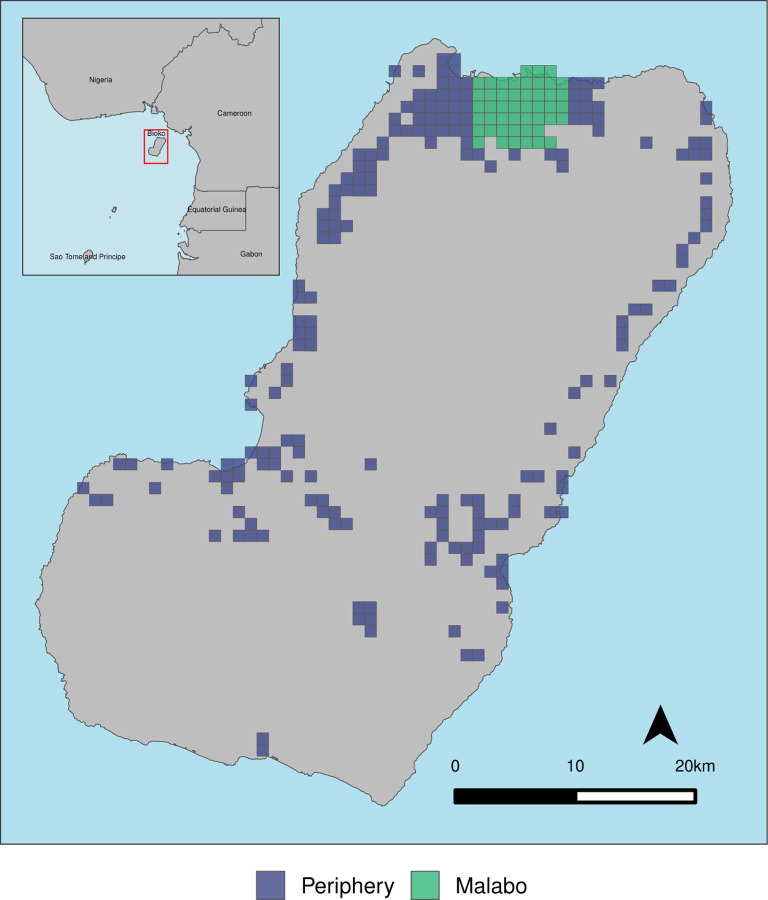
Bioko Island and its 1×1 km grid of inhabited areas. Areas are represented by the boxes [10] stratified by urban Malabo (47 areas) and the rural periphery (193 areas).

Bioko Island is the largest island of Equatorial Guinea and lies in the Gulf of Guinea off the coast of Cameroon (Figure 1). The island’s unique epidemiological profile, which is characterised by a high malaria burden and a dense population in the country capital, Malabo, offers both opportunities and challenges for vector control interventions. Since 2004, the Bioko Island Malaria Elimination Project (BIMEP), through the National Malaria Control Programme, has implemented a comprehensive malaria control package that includes improved diagnosis and treatment, intermittent preventive treatment for pregnant women, epidemiological and entomological surveillance, social and behaviour change communication (SBCC), and a particular emphasis on vector control through sustained indoor residual spraying (IRS), larval source management (LSM) and long-lasting insecticidal nets (LLINs).

LLIN distribution has gone through multiple iterations in a quest for optimal cost-effectiveness. During the first decade of the BIMEP the main vector control intervention was IRS implemented island-wide at high coverage. An early LLIN mass distribution campaign (MDC) took place in 2007, but it was not a consistent strategy at the time. In 2015, the overall vector control strategy shifted from IRS to triennial LLIN MDC backstopped by targeted IRS. Two MDCs across the whole island were conducted, one in 2015 and one in 2018. Both campaigns were supported by comprehensive SBCC activities aimed at boosting LLIN use and upkeep.

A door-to-door, top-up distribution campaign was implemented *ad hoc* in 2020 to support vector control in the middle of the SARS-CoV-2 pandemic. By 2021, following the triennial frequency recommendation, another island-wide MDC was to take place. However, the strategy instead shifted to restricting door-to-door MDC to rural areas and establishing fixed distribution points in urban Malabo. This modification aimed to streamline the distribution process, reduce logistical burdens, and better align with the population’s needs and behaviors.

### Assessing LLIN coverage through standard indicators

**Figure 2:**
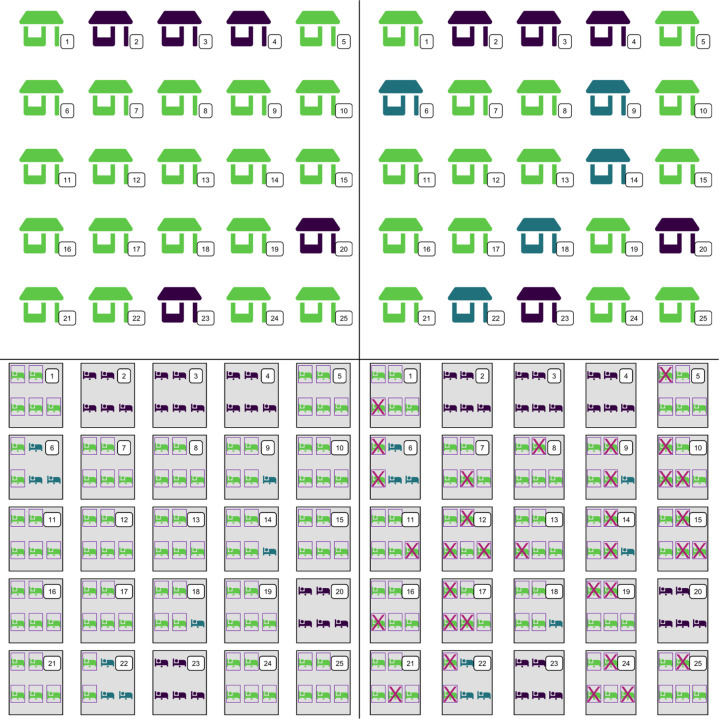
LLIN indicators. Illustration of how the four indicators are calculated using a simple schematic of an area (Figure 1) populated by 25 households, each with 5 residents. Households are enumerated sequentially. **Top left: Household ownership.** Green houses have at least one LLIN and purple houses have no nets (20/25, or 80%). **Top right: Household access.** Houses with at least one LLIN but with less than one LLIN for every two members are colored blue (15/25, or 60%). **Bottom left: Population access.** Sleeping residents colored in green and surrounded by a purple box have access to a LLIN within their home; individuals in blue inhabit a house with nets but do not have access to one and those in purple inhabit a house with no nets. Since one LLIN protects two people, households with all individuals colored green have three LLINs (*e.g*. houses 1, 5 and 25). Despite living in a house with less than one LLIN for every two occupants (*i.e*. blue houses in the top right panel, such as house 6 and 14), some residents still have access to a net. House 6, for example, has one LLIN to protect two residents. Population access in this example is 72.8% (91/125 people), higher than household access given that some occupants within households with not enough nets to serve all could still sleep under a net. **Bottom right: Use.** Sleepers with no access to a net (blue and purple) cannot use one. Some residents choose not to use a net despite having access to one (i.e. green sleepers marked with an X). LLIN use was 45.6% (57/125 residents). Among the 68 non-users living in the area, half of them lacked access while the other half chose to not use available LLINs. **U:A** is 45.6/72.8 = 0.63, or 63% of those with access used a net (57/91 residents). Source of infographics: Font Awesome by Dave Gandy - http://fontawesome.io.

LLIN coverage is a critical indicator for assessing the intervention. This measurement is captured through a series of standard indicators, each designed to shed light on different aspects of LLIN distribution and utilization within a community [1, 2, 3]. There are two household-level (*i.e*. ownership and access) and three individual-level indicators (*i.e*. population access, use and use-to-access; Table 1). Figure 2 illustrates how these indicators are calculated and highlights important interactions between them, critical for their interpretation.

*Ownership* gives an estimate of LLIN availability in the community, but it can overestimate access within the home. *Household access*, on the other hand, measures the proportion of households with sufficient nets to cover all their residents but ignores the fact that some members may still have access despite there not being enough LLINs to serve all occupants. The individual indicator of *population access* measures the proportion of individuals who are able to sleep under a LLIN by considering the availability of such nets within their households, thus rendering a more realistic estimate of access.

The effectiveness of LLINs hinges primarily on individual *use*, which determines both individual and community protection through several mechanisms [4]. Net users are protected by the physical barrier between them and host-seeking mosquitoes. Use of nets also exposes vectors to the insecticide, increasing mosquito mortality and reducing vector densities. Nets may also deter house entry by mosquitoes, further reducing blood feeding rates. Use is primarily conditional to having access to a LLIN, though despite high ownership and access rates, actual LLIN use might not align [3]. An important indicator, therefore, is the fraction of users among those with access, expressed as the *use-to-access ratio* (U:A) [3]. This metric is critical as there are important differences between non-users without access and non-users despite access.

### The challenges of universal coverage

Achieving universal LLIN coverage is challenging due to various factors [2]. First, LLIN distribution can be costly and logistically challenging depending on the strategy adopted [5]. Second, ensuring LLINs are distributed at the recommended ratio of one for every two people is challenged by inefficient distribution systems that tend to over-allocate LLINs to some households [6]. Third, rapid LLIN attrition post distribution affects access and sustained rates of use. Estimates show on average one-half of the LLINs distributed are lost two years after allocation [6, 7]. Finally, certain cultural and behavioral factors are critical determinants of LLIN use and deserve close attention and tailored distribution mechanisms [8].

This paper critically analyses LLIN coverage on Bioko Island through an examination of the standard LLIN indicators at fine spatial granularity. Due to the logistically complex and resource intensive nature of MDCs, particularly in densely populated urban areas, differences between the city of Malabo and rural Bioko were investigated (Figure 1). The analyses integrated data from distribution campaigns and annual malaria indicator surveys (MIS) conducted between 2015 and 2022. The 2021 and 2022 MIS were used to evaluate the effects of the shift in LLIN distribution strategy on coverage. The study aimed to help make LLIN distribution systems work better, not just on Bioko Island but also in other malaria-endemic areas. The paper also underscores the importance of adaptive management in malaria control programmes and the need for continuous, data-driven adjustments to maximise the public health benefits of malaria control activities.

## Methods

### Study area

The study comprised all inhabited areas of Bioko Island. Approximately 80% of the population of Bioko lives in Malabo and its surroundings. The island was stratified into urban *Malabo* and the, largely rural, *periphery* (Figure 1) using high resolution population data from the 2018 MDC [9, 10]. The stratification was based on the distribution of the population in urban Malabo and surroundings, whereby areas were aggregated to conform a continuum. Median population density in this stratum was 2,666 people/km^2^ compared to 54 people/km^2^ in the periphery stratum.

### Data

#### A spatial decision support system

The analyses leveraged the BIMEP’s spatial decision support system (SDSS), which is described in detail elsewhere [11, 9]. Briefly, the SDSS consists of a centralised database server, a geographic grid-based household coding system and a form-based data collection system. The grid-based system is constructed around a 1×1 km *areas* grid. Household intervention data are linked spatially through unique household codes making it possible to render LLIN indicators at very high spatial resolution. The analyses were based on the 240 inhabited areas: 47 corresponding to Malabo and 193 to the periphery (Figure 1).

#### LLIN distribution data

Data from MDC were linked to households to estimate the number of LLINs distributed per household. The 2015 and 2018 MDC took place between October 2014 and July 2015 and between February and November 2018. These campaigns targeted every household on the island, but could not reach those found closed or where residents would not accept the intervention. The 2021 MDC in the periphery had the same configuration as the previous campaigns and was conducted between mid June and mid July.

During each MDC distribution, data on numbers of individuals and sleeping spaces within each household, as well as numbers of LLINs owned and those delivered, were recorded. For every household, the total number of LLINs available after the distribution, *N*_*t*_, was estimated as follows:

$$N_{t}=N_{g}+N_{s}$$

where *N*_*g*_ is the number of pre-existing nets verified in good condition and *N*_*s*_ the number supplied during distribution. *N*_*t*_ was used to assess the change in the number of LLINs per household after each MDC.

#### MIS data

Annual MIS data between 2015 and 2022 were used to estimate LLIN indicators. MIS are conducted annually during August and September, in the second half of the rainy season, based on a representative sample of about 6% of the whole-island population [12].

Individual-level data were linked to the corresponding household, which in turn was linked to the SDSS geographical grid [9, 11]. Surveyed individuals were asked whether they slept under a LLIN the night before. Formally, estimates of use should be based on *de facto* household members (*i.e*. those who slept in the house). However, this information was captured from 2019 onwards only. For comparability across years, we estimated LLIN use based on *de jure* members (*i.e*. all household members).

MIS also enquire about the number of LLINs available at the household, or LLINs *declared* and abbreviated *N*_*d*_. Whenever possible, surveyors corroborated their presence by observing LLINs within the household. *N*_*d*_ was assumed an accurate reflection of the actual LLIN crop in each household. For each individual net, specific information is recorded, including the source. For 2021 and 2022, these data were used to categorise LLINs as those obtained at fixed distribution points or those procured elsewhere. This allowed comparison of LLIN use between the two source categories.

### LLIN indicators

All indicators were calculated as described in Figure 2 using *N*_*d*_ and LLIN use the previous night from the MIS. Standard survey data analysis techniques were applied to account for the sampling design, as follows. A two-level stratified cluster design was declared using primary sampling units (PSUs) and households as clusters. For each PSU, the sampling probability was set to 1 (i.e., a certainty PSU), and the household sampling probability was defined as the number of households sampled in a PSU divided by the number of inhabited households in that unit, according to a predefined sampling frame. In addition, a finite population correction was performed to account for the fact that all PSUs were sampled.

A target of 80% coverage was used as the reference for evaluating indicators against universal coverage. In the case of U:A, 80% corresponds to a value of 0.8. In addition to assessing indicators, LLIN attrition was measured by comparing *N*_*t*_ against *N*_*d*_ for households surveyed in MDC years (2015, 2018 and 2021). Finally, the impact of the new LLIN strategy in Malabo was evaluated using MIS data in 2021 and 2022. Since the questionnaires enquire about the source of nets, this was used to discriminate LLIN users who obtained their LLINs at the newly created fixed distribution points from those who obtained them elsewhere (*e.g*. previous MDC or ANC).

### Regional data

Data on LLIN indicators from other malaria endemic areas were obtained from the 2022 World Malaria Report [13]. These were used for contextualizing the Bioko Island LLIN indicators relative to those in other countries in sub-Saharan Africa.

### Data analyses

Weighted estimates, and their corresponding confidence limits, were generated for all indicators at area-level and for Malabo and the periphery. The association between population access against use and against U:A was investigated using generalised additive models to account for non-linear e ects on the response variable. The models were fit using weights based on MIS sample size. All analyses and all figures were coded in R [14].

## Results

### LLINs distributed since 2015

Table 2 summarises LLIN distribution data between 2015 and 2022. During this period, 537,583 LLINs were distributed, 74.8% of them (402,000) through door-to-door campaigns. During the 2015 MDC, 149,097 LLINs were supplied to 60,810 households, 84.9% in Malabo. In 2018, 63,149 households received 156,061 LLINs, 83.6% in Malabo. The 2021 MDC in the periphery distributed 30,558 LLINs to 11,308 households. The top-up, door-to-door distribution in 2020 provided 66,284 LLINs to 24,774 households, 79.4% in Malabo.

Continuous distribution in antenatal clinics has been part of the package for several years, though data were recorded consistently only since 2021 (4,886 nets in 2021 and 5,941 in 2022). In addition, a large school-based campaign took place in 2017, whereby 35,365 LLINs were distributed. Through the newly implemented distribution strategy, a total of 89,391 LLINs were delivered at six fixed distribution points in Malabo (53,489 in 2021 and 35,902 in 2022).

### LLIN coverage

**Figure 3:**
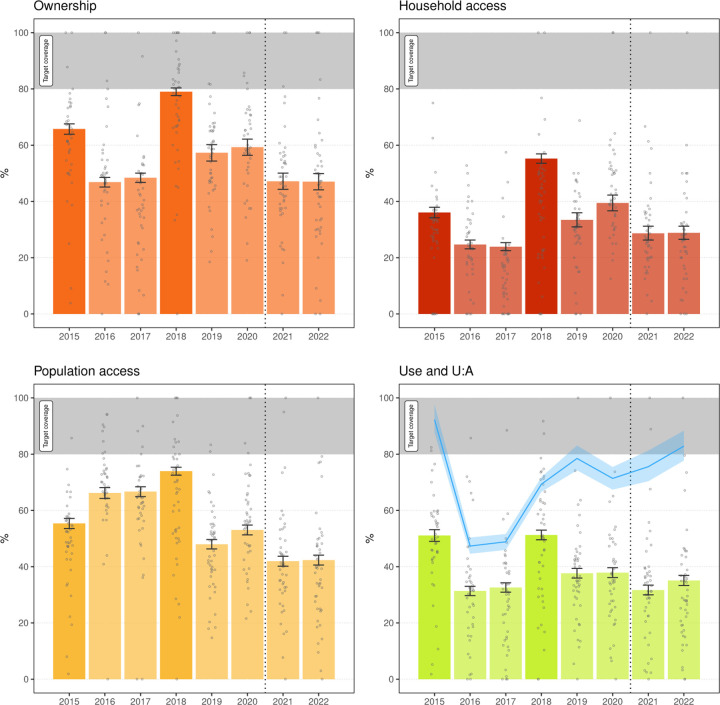
LLIN indicators in Malabo, 2015–2022. Bars and error bars mark the overall mean and 95% confidence intervals of survey weighted estimates. Bars with greater colour saturation indicate MDC years. The grey circles illustrate area-level weighted estimates for the 47 areas in Malabo. In the lower right panel, green bars and grey circles express LLIN use and the blue line the mean U:A with the shaded area corresponding to the 95% confidence intervals. The grey band marks the target universal coverage (≥ 80%). The vertical dotted line indicates the change in distribution strategy in Malabo.

**Figure 4:**
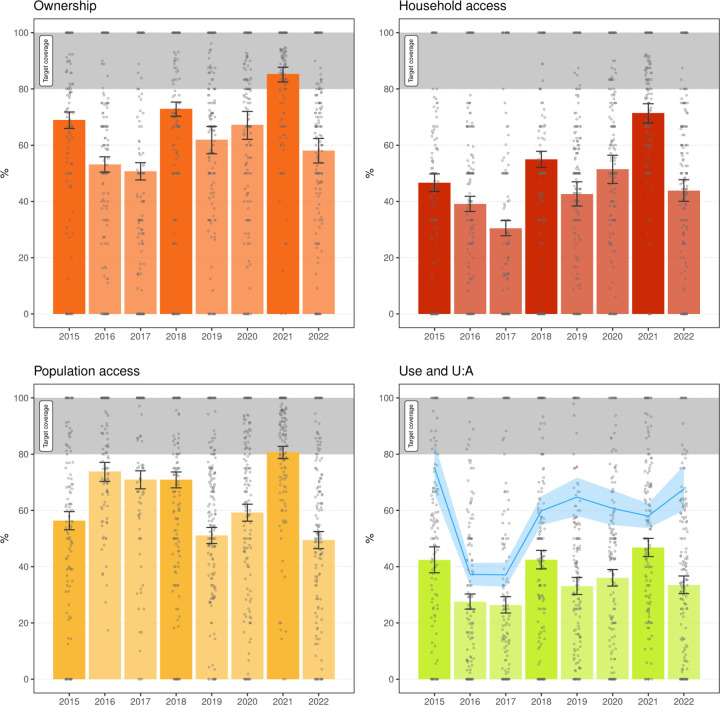
LLIN indicators in the periphery, 2015–2022. Bars and error bars mark the overall mean and 95% confidence intervals of survey weighted estimates. Bars with greater colour saturation indicate MDC years. The grey circles illustrate area-level weighted estimates for the 193 areas in the periphery. In the lower right panel, green bars and grey circles express LLIN use and the blue line the mean U:A with the shaded area corresponding to the 95% confidence intervals. The grey band marks the target universal coverage (≥ 80%).

**Figure 5:**
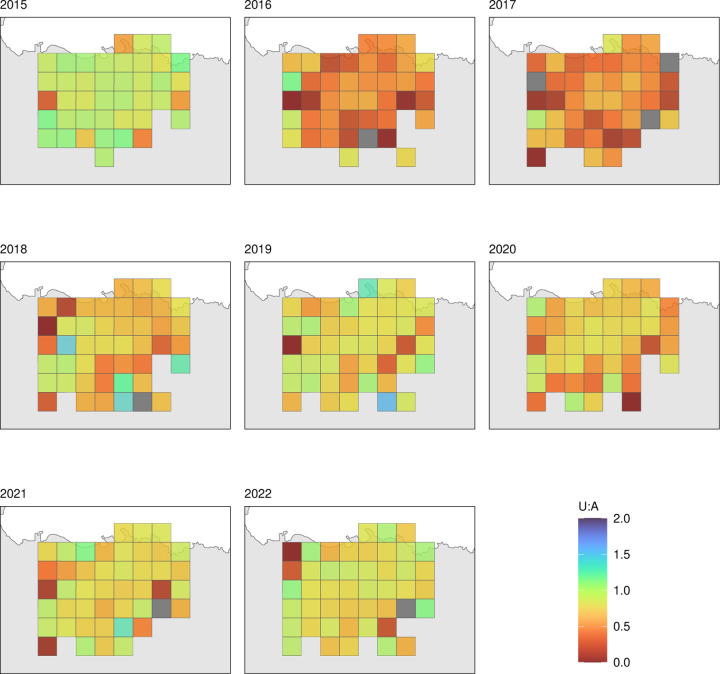
Spatial rendering of U:A in Malabo, 2015–2022. Only Malabo areas, as per Figure 1, surveyed in each year are plotted. MDC took place in 2015 and 2018.

**Figure 6:**
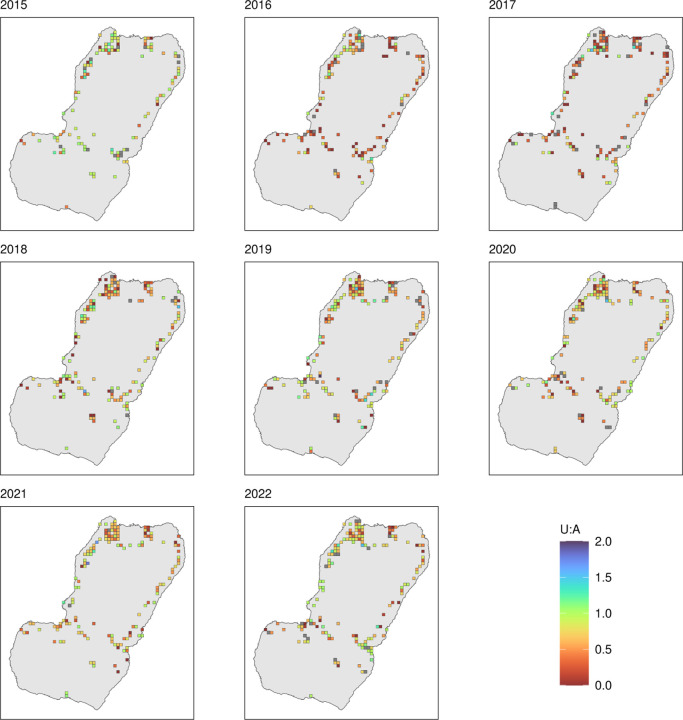
Spatial rendering of U:A in the periphery, 2015–2022. Only areas in the periphery, as per Figure 1, surveyed in each year are plotted. MDC took place in 2015, 2018 and 2021.

Figures 3 and 4 illustrate LLIN indicators for Malabo and the periphery. A critical result was the very important variability of all indicators over time across areas. This variation was more evident in the periphery, where area-level sample sizes are comparatively small, which could partially confound this observation. Figures 5 and 6 illustrate this heterogeneity for U:A.

Between 2015 and 2020, all indicators reflected similar coverage in Malabo and the periphery, with spikes during MDC years, particularly noticeable in ownership, followed by troughs in years between. As expected, household access was significantly lower than ownership. After the 2015 MDC, ownership, household access and use dropped significantly in 2016 and 2017. Conversely, population access increased in those years relative to 2015 in both strata, possibly due to LLIN redistribution amongst households. This observation, together with the drop in use rates in 2016 and 2017, explains the substantial decrease in U:A. The 2018 MDC boosted all indicators and was followed by another significant drop in 2019, which was reverted in 2020, probably explained by the top-up distribution. A similar trend was not observed in LLIN use, which remained under 40% in both Malabo and the periphery.

In 2021 in the periphery, the MDC significantly boosted ownership and population access, with both crossing the 80% mark, as well as household access and use. U:A, however, dropped, evidencing a large gap between population access and use. The gains achieved in 2021 were not sustained, and by 2022 all indicators had significantly fallen again. In Malabo, coverage according to all indicators decreased in 2021 relative to 2020. In 2022, however, there were no further drops in coverage, and U:A increased since 2020 and surpassed 80% in 2022.

Despite relatively high population access across all years in both strata, use was persistently low, with a mean of 38.6% and 36.0% people reportedly using a LLIN in Malabo and the periphery, respectively. U:A showed significant variation over the years. In 2015, despite low overall use, U:A was at its highest with an average 92.3% and 75.2% of people with access using a LLIN in Malabo and the periphery, respectively. This was driven by the relatively limited population access observed post MDC in that year. In 2016 and 2017, U:A dropped significantly to less than 40% in the periphery and less than 50% in Malabo, before increasing to between 60 and 70% in the periphery and to over 70% in Malabo, where, in 2022, reached 82.9%.

### LLIN attrition post MDC

Table 3 summarises LLIN attrition by comparing *N*_*t*_ with *N*_*d*_ in MDC years. In 2015, out of 5,160 households surveyed in the MIS, 4,463 (86.5%) had at least one *N*_*t*_ after the MDC in that year. Almost half the nets distributed were not declared (44.0% in Malabo and 41.1% in the periphery). Households were surveyed a mean of 5.4 and 3.5 months post MDC, respectively. In 2018, amongst the 4,762 households surveyed, 4,186 (87.9%) had at least one *N*_*t*_ after MDC distribution. A lower proportion of nets were not declared four and three months after the MDC in Malabo (31.6%) and the periphery (27.2%). In 2021, 1,768 households were surveyed in the periphery, of which 1,682 (95.1%) had at least one *N*_*t*_ after the MDC, with 23.1% of LLINs distributed not declared in the MIS at a mean of 1.4 months after distribution.

### Relationship between indicators

**Figure 7:**
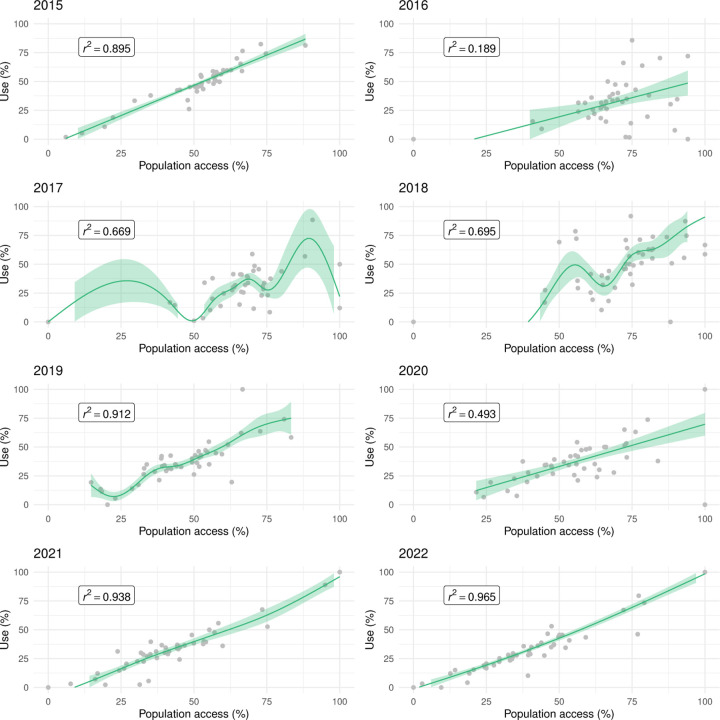
The relationship between LLIN use and population access at area level in Malabo. The line and shaded areas represent GAM fits and credible intervals.

**Figure 8:**
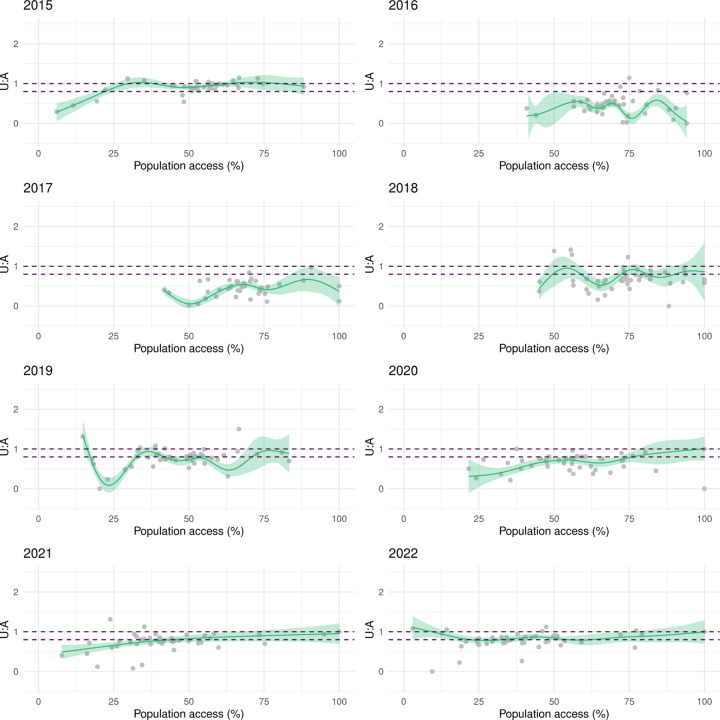
The relationship between LLIN U:A and population access at area level in Malabo. The line and shaded areas represent model fits and credible intervals. The dashed lines represent the band of universal coverage (80–100% U:A).

Figure 7 illustrates the relationship between LLIN use and population access in areas of Malabo. There was a strong, positive, mostly linear relationship in 2015, 2019, 2021 and 2022. In 2020, the relationship was linear but weaker. In 2016, 2017 and 2018, use rates were highly heterogeneous amongst areas, with use lower than 50% in many despite good access. In these years, U:A remained mostly under the 80% target, as well as in 2020, when U:A showed some improvement with increasing access (Figure 8)

**Figure 9:**
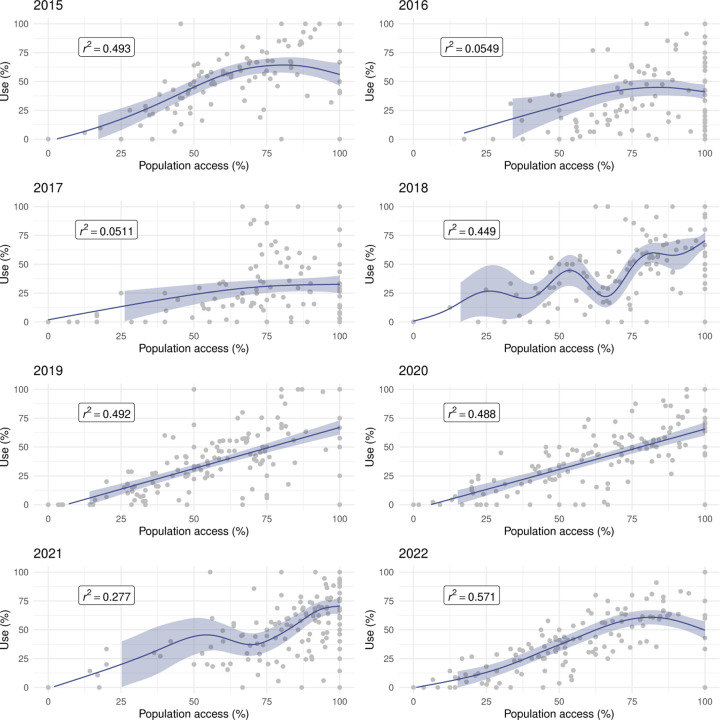
The relationship between LLIN use and population access at area level in the periphery. The line and shaded areas represent GAM fits and credible intervals.

**Figure 10:**
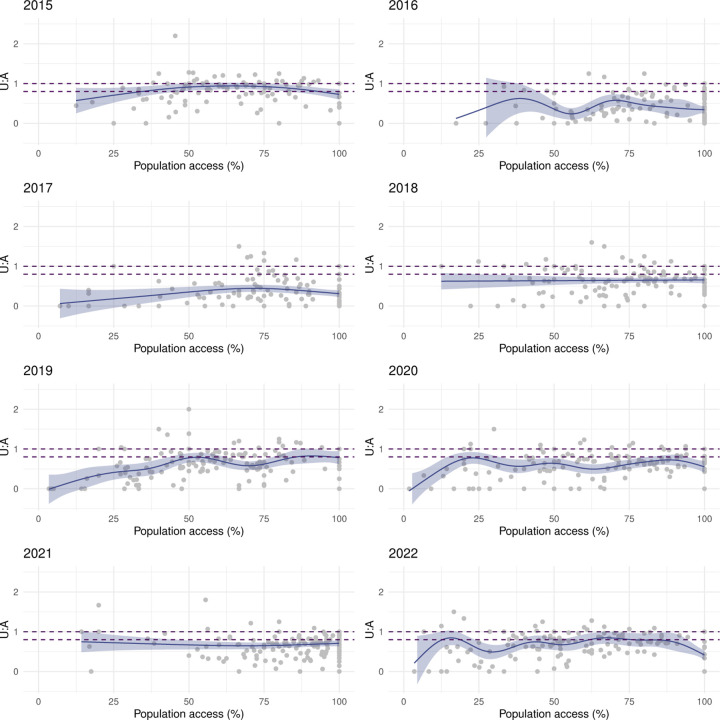
The relationship between LLIN U:A and population access at area level in the periphery. The line and shaded areas represent model fits and credible intervals. The dashed lines represent the band of universal coverage (80–100% U:A).

In the periphery, the relationship between use and population access was weaker even during MDC years (Figure 9). Areas showing sub optimal use rates despite universal population access (≥ 80%) were commonplace, which was reflected in the model outputs as plateaus and dips in use when very high access was observed. With the exception of 2015, U:A modelled estimates remained lower than the target and, in most years, dropped at very high access (Figure 10), revealing a counter-intuitive relationship.

### LLIN indicators after the change in distribution strategy

Figure 3 reveals a significant drop in population access in Malabo in 2021 relative to 2020. The door-to-door top-up campaign in 2020 covered large portions of Malabo and can explain the high population access in 2020 followed by attrition. Conversely, population access in 2022 was sustained relative to 2021. In 2021, use dropped relative to 2020 but increased again in 2022. Critically, U:A in Malabo increased in 2021 and surpassed the 80% target in 2022. This was not followed in the periphery, despite the 2021 MDC.

**Figure 11:**
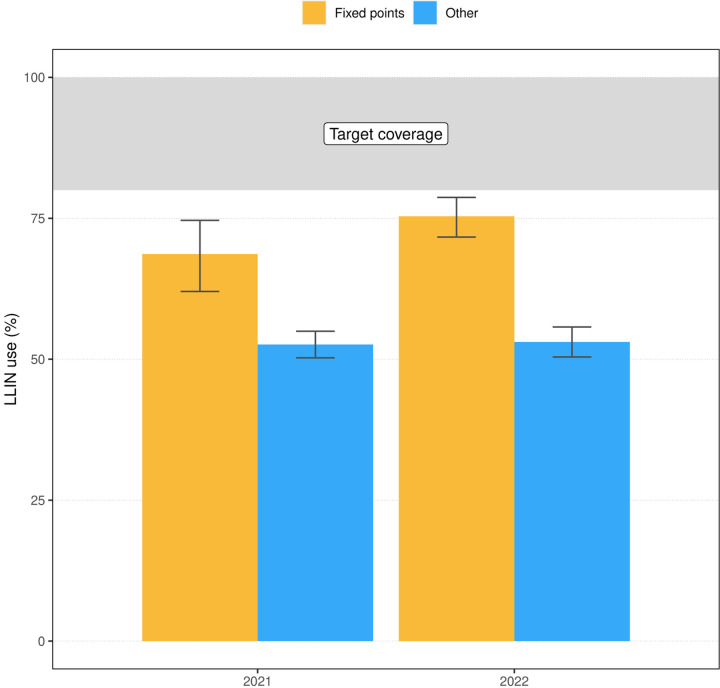
LLIN use in Malabo in 2021 and 2022 according to source of the nets. LLIN use amongst households with access (*i.e*. at least one net for every two members) and with at least one net sourced from fixed distribution points (orange) or from other sources (blue), by year. Error bars represent the 95% confidence intervals of the estimates.

Individual MIS data allowed further investigation of LLIN use amongst those who sourced their nets at the fixed distribution points in 2021 and 2022. Figure 11 shows LLIN use was significantly higher in households where LLINs were obtained from a fixed point both in 2021 (68.7%) and 2022 (75.4%) compared to those where nets came from other sources (52.6% in 2021 and 53.1% in 2022). The percentage of households with LLINs from fixed distribution points was low in 2021 (6.1%) but increased significantly in 2022 (19.6%), which can explain the concurrent improvement in U:A in the latter year.

### Regional comparisons

**Figure 12:**
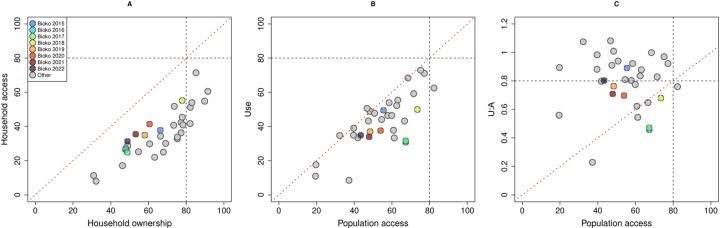
The relationship between LLIN indicators on Bioko Island compared to data from 25 other malaria endemic countries in sub-Saharan Africa [13]. (**A**) Household access against ownership; (**B**) use against population access and (**C**) U:A against population access. The black dotted lines represent the thresholds for universal coverage for each indicator in the *x* and *y* axes. The red dotted diagonals mark equity between indicators.

In Figure 12, the relationship between LLIN indicators on Bioko Island is presented relative to indicators obtained for other sub-Saharan African countries [13]. Universal coverage was achieved only in a quarter of these countries according to ownership, in one according to population access and in 17 according to U:A, suggesting that population use in the latter could be limited by lack of access [3]. Estimates on Bioko revealed universal coverage could not be sustained even in MDC years. With the exception of 2015, when 89.1% of people with access reported using a LLIN, U:A between 2016 and 2021 was comparatively lower than as reported in other countries. In 2022, U:A was 80.3%, mostly driven by the high U:A achieved in Malabo.

## Discussion

The significant challenges of achieving universal LLIN coverage faced by malaria control programmes are reflected in the persistently sub-optimal indicators reported across malaria endemic sub-Saharan Africa. In 2018, despite 72% of households in the region had at least one net, only 40% of the population lived in households with enough nets for all occupants and only 57% had access to a net within their home (*i.e*. population access). In the same year, mean LLIN use was estimated at 50%, a significant leap from the 29% estimated in 2010, but still short of the desired universal coverage target [1]. Notwithstanding the considerable efforts invested in guaranteeing population access and recent evidence confirming use is significantly associated with a reduction in the odds of local malaria infection [15], Bioko continues to face similar challenges, with sub-optimal LLIN population access and poor use rates (median of 54.7 and 35.9%, respectively, between 2015 and 2022). U:A was closer to the target, with an overall median of 0.76, indicating most people with access to a net within their home used it. This estimate, however, was lower than other countries in the region where a mean U:A of 0.83 has been reported (Figure 12C). In terms of LLIN retention time, a recent estimate put Equatorial Guinea high on the rank at 3.6 years [16], suggesting the population could cope with triennial MDC. One problem with this estimate, however, is that it glosses over spatial variability.

Indeed, a striking finding of our work was the considerable spatial heterogeneity observed in indicators. Even though LLIN use was positively correlated with population access, particularly in Malabo, in many areas of the island getting people to keep and use their nets after MDC appeared a major hurdle towards achieving high LLIN coverage (Figures 5 and 6). Area-level estimates of population access showed significant drops soon after MDC. Given that MDC targeted all accessible households, this heterogeneity is a reflection of the great variation in behavioural and environmental factors rather than of actual lack of access to nets. First, there is the inherent heterogeneity in the perceived and actual risk of malaria transmission, which can drive people to want and use a LLIN or not [17]. Second, there is considerable heterogeneity in housing characteristics [15], which determines the risk of being bitten indoors as well as the predisposition to bear discomfort when of sleeping under a net [17]. Some houses are built with cement walls and roofs, some with precarious wood walls and tin roofs; some have open eaves, some have closed eaves; some have access to clean water, others do not and need to collect it favoring mosquito breeding in their neighborhoods; some households use air conditioning, some do not. Finally, there are cultural and socioeconomic factors that determine some people are more prone to adhering to malaria interventions than others [18]. Adherence is one critical driver for the success of any intervention and this also varies across areas of Bioko Island.

MDCs are a resource intensive strategy. This is particularly the case in densely populated urban areas, such as Malabo. To provide context, the island-wide 2015 and 2018 MDC took around six months to implement with a workforce of about 90 people. By contrast, the 2021 MDC restricted to the periphery demanded one month and required the effort of only 16 workers. Moreover, during the 2015 and 2018 campaigns, LLINs were hung over sleeping spaces to further motivate the population to use them, but this seemed to have made little difference on indicators. Low LLIN use and poor retention observed in the MIS data motivated the shift in distribution strategy in the city towards fixed points, justified on the grounds of poor gains obtained relative to the great efforts invested in MDCs. The rationale behind this shift was to optimise resources while guaranteeing LLIN access to the people who use them. Data from the 2021 and 2022 MIS suggested those who obtained their nets from distribution points showed significantly higher use rates than those who did from other sources (Figure 11).

Given the novelty of this modality of distribution, however, only a small fraction of households surveyed in 2021 reported obtaining the nets from fixed distribution points (6.1%). The substantial leap in 2022 to a fifth of households sourcing their nets in these facilities is encouraging. The relatively low proportion of the population attending distribution points can explain the lack of improvement in population use and access in Malabo in 2021 and 2022 (Figure 3). On the other hand, U:A did show a significant improvement in these years, further supporting the strategy. Efforts must be devoted towards reinforcing SBCC to raise population awareness of where LLINs are available whenever they need them.

The question of what is the optimal distribution strategy for the periphery remains unanswered. On the one hand, data showed that, in sharp contrast to what was observed in Malabo, population access significantly dropped in 2022 in the periphery following the 2021 MDC (Figure 9). On the other hand, as mentioned above, deploying MDC in the periphery is substantially less onerous than in urban Malabo while operating fixed distribution points could prove logistically more difficult to manage across larger distances. It is also important to note that malaria transmission in parts of the periphery is significantly higher, begging for more targeted distribution that both optimises efficiency while serving households at higher risk and more likely to keep and use LLINs. This would be based on an assessment of housing characteristics and other metrics such as socioeconomic status [15]. Continuous monitoring of LLIN indicators will shed light on how best to tailor the intervention.

Guaranteeing population access is the first step towards closing the net use gap [3]. Many programmes continue to rely on MDCs every two to three years to provide their populations with high LLIN coverage. In 2022, 44 countries planned such campaigns to distribute 241 million LLINs, which required spending very significant logistical and financial efforts [19]. While MDCs help improve ownership, population access and use of nets immediately after distribution [2, 20, 21, 22, 23, 24, 25], these endeavours often deteriorate into inefficient systems with nets unevenly distributed amongst households [6]. Furthermore, LLIN indicators frequently drop steeply relatively soon after distribution compromising use, as observed on Bioko [26, 27]. Recent momentum is building towards improving efficiency through alternative continuous distribution channels [16, 5].

The evidence presented here supports the adoption of new mechanisms for getting LLINs to the population, and fixed distribution points seem a plausible option. The strategy in Malabo prioritises giving nets to those members of the population who need them and who would use them. This new strategy had the additional benefit of cost effectiveness. Not only it improved coverage measured through use and U:A, but sustaining the fixed distribution points saved the programme considerable resources, with an estimated cost less than half that of triennial whole-island MDC. Crucially, the resources released are being invested in alternative interventions to tackle residual biting that cannot be averted by the use of nets, such as expanding larval source management in Malabo.

Beyond the need of adapting distribution strategies, what is also clear from the analyses in this paper is that the net use gap is both substantial and heterogeneous across Bioko. The highly granular spatial heterogeneity of LLIN indicators probably operates in many other settings but is difficult to account for. The spatially resolved data of the Bioko SDSS has allowed to capture the nuances behind LLIN access and use and leverage these to adapt strategies. Furthermore, these nuances revealed that narrowing the net use gap requires a lot more than guaranteeing access and calls for better tailoring SBCC in order to improve adherence and sustain access. Part of adaptively managing LLIN strategies is about intuitively finding the best means and messages to reach the population. This is another fundamental advantage of the SDSS package on Bioko, as, like any other intervention, communication strategies need to respond to data.

## Figures and Tables

**Table 1:** LLIN indicators and their definitions.

Indicator	Level	Standard definition	Comments
**Household ownership**	Household	Percentage of households that own at least one LLIN in good condition.	Here referred to as *ownership*. While it gives a general sense of coverage, it also fails to reflect actual availability of LLINs for the population.
**Household access**	Household	Percentage of households that own at least one LLIN in good condition for every two household members.	Does a better job at indicating actual availability of nets but does not account for households that have LLINs to protect at least part of their members.
**Population access**	Individual	Percentage of the population with access to LLINs within their home, assuming that one LLIN protects two people.	Preferred indicator to measure actual access to LLINs.
**Use**	Individual	Percentage of individuals who sleep under a LLIN.	Best measured among *de facto* household members, or the individuals who slept in the house the previous night.
**Use-to-access ratio (U:A)**	Individual	The ratio of use to access reflects the proportion of individuals who sleep under a LLIN provided they have access to one.	A U:A ratio of 1 means that all residents who have access to LLINs are using them; a U:A < 1 suggests that residents are under-utilizing LLINs despite having access; a U:A > 1 indicates that LLINs available are used by more than two people [2, 3]. High coverage is defined by U:A ≥ 0.8, denoting ≥ 80% LLIN usage among those with access.

**Table 2:** Number of LLINs distributed on Bioko Island between 2015 and 2022 through different channels.

Year	High risk groups	Door-to-door	Fixed points	Total
2015	-	149,097	-	149,097
2016	-	-	-	*
2017	35,365	-	-	35,365
2018	-	156,061	-	156,061
2019	-	-	-	*
2020	-	66,284	-	66,284
2021	4,886	30,558	53,489	88,933
2022	5,941	-	35,902	41,843
**All years**	46,192	402,000	89,391	**537,583**

**Table 3:** LLIN attrition post MDC.

Year	Stratum	*N*_*t*_	*N*_*d*_	LLINs lost (%)	Months since MDC
2015	Periphery	3,429	2,021	41.1	3.5
	Malabo	9,194	5,145	44.0	5.4
2018	Periphery	4,129	3,006	27.2	3.1
	Malabo	10,176	6,962	31.6	4.0
2021	Periphery	5,613	4,317	23.1	1.4

## Data Availability

The datasets used and/or analysed during the current study are available from the corresponding author on reasonable request.
